# The Therapeutic Potential of Anti-Inflammatory Exerkines in the Treatment of Atherosclerosis

**DOI:** 10.3390/ijms18061260

**Published:** 2017-06-13

**Authors:** Megan Yu, Sheng-Feng Tsai, Yu-Min Kuo

**Affiliations:** 1 Department of Chemistry, University of Virginia, Charlottesville, VA 22904, USA; 2 Institute of Basic Medical Sciences, National Cheng Kung University, Tainan 70101, Taiwan; eric04142000@hotmail.com (S.-F.T.); kuoym@mail.ncku.edu.tw (Y.-M.K.); 3 Department of Cell Biology and Anatomy, National Cheng Kung University, Tainan 70101, Taiwan

**Keywords:** atherosclerosis, exerkine, physical exercise

## Abstract

Although many cardiovascular (CVD) medications, such as antithrombotics, statins, and antihypertensives, have been identified to treat atherosclerosis, at most, many of these therapeutic agents only delay its progression. A growing body of evidence suggests physical exercise could be implemented as a non-pharmacologic treatment due to its pro-metabolic, multisystemic, and anti-inflammatory benefits. Specifically, it has been discovered that certain anti-inflammatory peptides, metabolites, and RNA species (collectively termed “exerkines”) are released in response to exercise that could facilitate these benefits and could serve as potential therapeutic targets for atherosclerosis. However, much of the relationship between exercise and these exerkines remains unanswered, and there are several challenges in the discovery and validation of these exerkines. This review primarily highlights major anti-inflammatory exerkines that could serve as potential therapeutic targets for atherosclerosis. To provide some context and comparison for the therapeutic potential of exerkines, the anti-inflammatory, multisystemic benefits of exercise, the basic mechanisms of atherosclerosis, and the limited efficacies of current anti-inflammatory therapeutics for atherosclerosis are briefly summarized. Finally, key challenges and future directions for exploiting these exerkines in the treatment of atherosclerosis are discussed.

## 1. Introduction

Atherosclerosis and its clinical manifestations in coronary heart disease, stroke, and peripheral artery disease are the leading cause of morbidity and mortality in the Western world [1]. Understanding the pathophysiology of atherosclerosis and developing potential means of treating the disease are of utmost importance, as it currently accounts for 17.3 million deaths and is expected to increase to more than 23.6 million by 2030 [2]. Although the etiology of atherosclerosis is complex, its development can be characterized into five stages: (1) low-density lipoprotein (LDL) retention in the arterial wall, (2) LDL oxidation and modification, (3) inflammation, (4) formation of foam cells and fibrous plaques, and (5) calcification, plaque rupture, and thrombosis. Briefly, following a high-fat, high-cholesterol diet, lipoproteins and their aggregates accumulate in the subendothelial layer of the arterial wall, followed by the generation of oxidized species that recruit monocytes and T-cells to the vessel wall. These monocytes then transmigrate into the intima, convert into macrophages, and take up lipoproteins, forming foam cells that eventually form fatty streaks—the hallmark of early atherosclerosis [3]. Some of these fatty streaks accumulate smooth muscle cells (SMCs), which secrete fibrous elements that form plaques made of connective tissue. These plaques are eventually substituted with collagen-rich fibrous tissue that undergoes matrix production and degradation, and form fibrous caps that may rupture, calcify, and cause thrombosis [4,5,6].

Atherosclerosis is now primarily considered a chronic inflammatory disease, as the vascular wall hosts many complex chronic inflammation events among various inflammatory molecules [7]. In addition, unstable atherosclerotic plaques are characterized by high levels of inflammatory cells and mediators, including leukotrienes, active proteases, and cytokines [8]. Because approximately two-thirds of heart attacks and strokes are caused by the rupture of an unstable atherosclerotic plaque [8], many anti-inflammatory drugs, such as statins, antithrombotics, and antihypertensives, have been developed to reduce these adverse events. However, these drugs at most only delay the progression of atherosclerosis. Due to its pro-metabolic, multisystemic, and anti-inflammatory benefits, physical exercise has been prescribed as a non-pharmacologic treatment for atherosclerosis, as it boosts high-density lipoprotein (HDL) levels while lowering LDL levels, and reduces the risk of many chronic diseases, including type 2 diabetes and cancer. It also has a number of anti-inflammatory effects, including reducing visceral fat, increasing levels of nitric oxide (NO), and secreting anti-inflammatory exerkines from skeletal muscles and other secretory organs.

Ever since Bente Pedersen and colleagues proposed that contracting skeletal muscle could function in an endocrine-like manner by releasing certain muscle-derived peptides, metabolites, and circulating RNA species (myokines), there has been increasing interest in identifying and exploiting their therapeutic potential [9]. Later, it has been found that these circulating cytokines and humoral factors do not merely originate from skeletal muscle; they are also secreted by adipose tissue (adipokines), the liver (hepatokines), and other secretory organs. Therefore, Mark Tarnopolsky and colleagues coined the term “exerkines” in 2016 to collectively describe these circulating species that are produced and secreted by any tissue or organ into the circulation and could facilitate the multisystemic benefits of exercise [10]. These exerkines are found in extracellular vesicles that either bud outwardly from the plasma membrane (microvesicles; 100–1000 nm in diameter) or originate from the exocytosis of multivesicular bodies (exosomes; 20–140 nm in diameter) [10]. Our focus of this review is to highlight major anti-inflammatory exerkines that could serve as potential therapeutic targets for atherosclerosis. We also briefly summarize the anti-inflammatory, multisystemic effects of exercise, the basic mechanisms of atherosclerosis, and the limited efficacies of current anti-inflammatory therapeutics for atherosclerosis to provide some context and comparison to the therapeutic potential of these anti-inflammatory exerkines. Finally, we discuss key challenges and future directions for deploying these exerkines in the clinic. Understanding the role of exercise and exerkines in the prevention of atherosclerosis may lead to future development of bioengineered targets in the treatment of atherosclerosis.

## 2. Basic Mechanisms of Atherosclerosis

### 2.1. LDL Retention in Arterial Wall

Atherosclerotic development usually begins with LDL accumulation in the subendothelial matrix of the artery. Seminal findings that atherosclerosis is induced by LDL receptor mutations provide strong evidence that elevations in LDL levels are sufficient to initiate atherosclerosis [11,12]. Lesions usually form in regions with arterial branching or curvature, as they have disturbed flow, a high oscillatory sheer index, and low endothelial shear stress [12]. Endothelial cells in atherosclerosis-resistant regions have an ellipsoidal structure and nuclear morphology and coaxial alignment in the primary flow direction whereas those in atherosclerosis-prone regions do not display an orderly pattern and have a cuboidal shape [13]. Endothelial cells exposed to atheroprotective or atheroprone regions may also “mark” their DNA, creating unique flow-dependent epigenetic landscapes [13,14]. Some biomarker candidates that could facilitate the mechanotransductive property of endothelial cells include platelet and endothelial cell adhesion molecule 1 (PECAM-1) in complex with vascular endothelial cadherin (VE-cadherin) and vascular endothelial growth factor 2 (VEGFR2) [15], G protein-coupled S1P1 (S1P receptor-1) [16], Piezo1 [17], and syndecan 4 [18]. LDL diffuses passively through endothelial cell junctions, and the interaction between LDL constituent apolipoprotein B (ApoB) and matrix proteoglycans seem to permit its retention in the artery. Other ApoB-containing lipoproteins, such as lipoprotein(a), also accumulate in the intima and appear to be particularly atherogenic due to their effects on angiogenesis, fibrinolysis, and SMC growth [19,20,21].

### 2.2. LDL Oxidation and Modification

As LDL is trapped in the arterial wall, arterial cells release oxidative products that can induce lipid oxidation. This process is thought to occur in two steps. First, lipids in LDL are oxidized with little change in ApoB. Then, when monocytes are recruited to the lesion and transform into macrophages, the protein portion of LDL is modified while the lipids in LDL continue to be oxidized, causing a loss of recognition by the LDL receptor [22]. This change causes cellular uptake of LDL by receptors that are not regulated by cholesterol levels, leading to an accumulation of cholesterol-loaded cells, or foam cells [23], that further promotes inflammation by inducing monocytes and lymphocytes but not neutrophils that bind to endothelial cells.

Trapped LDL also undergoes other modifications that contribute to inflammation and foam cell formation, such as lipolysis, proteolysis, and aggregation [24]. Unlike LDL, it was originally thought HDL could prevent atherosclerosis due to its anti-oxidative, anti-inflammatory, antithrombotic, and profibrinolytic effects and by removing excess cholesterol from peripheral tissues inhibiting lipoprotein oxidation [25]. These effects are mainly caused by serum paraoxonase, an esterase on HDL that can degrade certain biologically active oxidized phospholipids [26]. However, recent findings have suggested HDL is impaired in patients with diabetes, coronary disease, chronic renal insufficiency, CVD risk factors and disorders, and patients who experienced coronary angiography but not in healthy individuals [27,28,29,30,31,32,33,34]. Specifically, the inverse correlation between HDL levels and the risk of future CVD events in healthy individuals may not apply and may, in fact, be abolished. HDL from these patient populations has also been found to have no protective vascular effects or could even cause paradoxical harmful effects [34]. This may be caused by significant changes in their pathological structure in an acute-phase reaction, during which HDL is characterized by increased concentrations of serum amyloid A, type 2 secretory phospholipase A2, and ceruloplasmin, and lower concentrations of apolipoprotein A1 [33].

### 2.3. Inflammation

Mild LDL oxidation causes monocyte and lymphocyte recruitment to the arterial wall by stimulating extracellular cells to produce several pro-inflammatory adhesion molecules and monocyte activators, such as macrophage colony-stimulating factor (M-CSF), monocyte chemoattractant protein 1 (MCP-1), and growth-related oncogene (GRO) [35]. In mice studies, Ly6^hi^ monocytes most readily enter developing atherosclerotic lesions, but atherosclerosis is maximally inhibited only when Ly6^hi^ and Ly6^lo^ monocytes are blocked [36]. Steps that enhance the proliferation of bone marrow-derived hematopoietic stem cells, such as cholesterol accumulation from defective cholesterol efflux, increase circulating monocytes and promote atherosclerosis [13,37]. Through a G protein-mediating mechanism, mildly-oxidized LDL elevates cAMP levels that decrease the expression of endothelial cell leukocyte adhesion molecule-1 (ELAM-1), and increase the expression of M-CSF, MCP-1, and GRO [38]. This process increases gene transcription rates and stabilizes the mRNA of these genes. Leukocyte adhesion is mediated by selectins that bind to carbohydrate ligands on leukocytes, and several adhesion molecules, including intercellular adhesion molecule-1 (ICAM-1), P-selectin, E-selectin, platelet endothelial cell adhesion molecule-1 (PCAM-1), and vascular cell adhesion protein 1 (VCAM-1), are critical for their recruitment [39]. Inflammasome activation is also found in atherosclerotic lesions, which involves cholesterol microcrystals from the cellular ingestion of retained lipoproteins and interleukin-1β (IL-1β) [40,41].

Oxidized LDL could also inhibit endothelin expression and nitric oxide (NO) production. This results in vasodilation reduction, as NO has been shown to be anti-atherogenic [42].

### 2.4. Formation of Foam Cells and Fibrous Plaques

Before being taken up by macrophages to form foam cells, LDL must be highly oxidized. This process involves reactive oxygen species (ROS) and enzymes that produce highly reactive species and promote LDL oxidation. For instance, myeloperoxidase generates hypochlorous acid and tyrosyl radicals while sphingomyelinase promotes lipoprotein aggregation. Secretory phospholipase (group II sPLA2) also promotes LDL oxidation [43,44]. Oxidized LDL is then recognized by macrophage scavenger receptors, such as SR-A, CD-36, and CD69, leading to foam cell formation [45]. These foam cells often accumulate in the proteoglycan layer of the intima and often appear as yellow-colored xanthomas or fatty streaks.

While many fatty streaks do not progress further, some become worse over time, especially those at predilection sites. In some areas, acellular, lipid-rich material accumulates in the intima without disrupting its structure. In other areas, the lipid pools grow into a large mass of extracellular lipid and promote the accumulation of SMCs and its extracellular matrix that contribute to the necrotic core of the intima. This process leaves a pool of lipids behind, such as cholesterol esters from LDL, free cholesterol-rich erythrocyte membranes from intraplaque hemorrhages, and cell debris devoid of a matrix [46]. Over time, the arterial intima is replaced with collagen-rich fibrous tissue, which is mainly produced by SMCs containing numerous rough endoplasmic reticulum and Golgi complexes. These SMCs are unique as they have undergone phenotypic modulation to the synthetic phenotype, and they accumulate substantially during lesion development [47,48]. They also downregulate expression of differentiation marker genes, such as those encoding muscle α-actin (Acta2) and smooth muscle myosin heavy chain (Myh11), thus increasing their production of extracellular matrix, proteoglycans, and proteins that are beneficial in outward vessel remodeling and plaque stabilization [13,49].

Circulating progenitor cells, multipotent stem cells in the media or adventitia, and synthetic SMCs in the arterial media are other possible sources of plaque SMCs [50]. However, some of these findings have been contested, and more research is needed to investigate the origin of plaque SMCs.

### 2.5. Calcification, Plaque Rupture, and Thrombosis

With increasing age and time, atherosclerotic lesions could result in calcification. Apoptotic cells, extracellular matrix, and necrotic core material are common sources of these calcification sites, and these sites can expand to form larger lumps of calcium deposits over time [31]. Many of these plaques often exclusively comprise of fibrous and calcified tissue without extracellular lipid pools or a necrotic core [51]. Plaque necrosis has been found to result from lesional macrophage apoptosis and defective efferocytosis, leading to post-apoptotic necrosis, loss of efferocytosis-mediated anti-inflammatory signaling, and generation of damage-associated molecular patterns (DAMPs) [13]. Receptor-interacting protein 3 (RIP3)-mediated necrosis may also play a role in this process [52]. Advanced lesional macrophage apoptosis is likely induced by many factors, such as oxidized lipoproteins, oxidized phospholipids, and accumulation of lipoprotein-derived cholesterol in the ER. Both macrophage apoptosis and defective efferocytosis are currently thought to result from compromised expression of mediators of atherosclerotic lesions and the amplification of major inflammatory events in atherosclerosis [13]. For example, the MerTK tyrosine oncogene, which is critical in lesional efferocytosis, could be cleaved when triggered by inflammation, thus possibly contributing to defective efferocytosis in advanced lesions [13].

Over time, some of these plaques could rupture and cause thrombosis. These ruptures often occur in the presence of a structural defect in the fibrous cap that is induced by inflammation, as these defects could cause pulsatile changes in arterial blood pressure. Plaque ruptures often occur at the edges of lesions, which are rich with foam cells, suggesting they contribute not only to inflammation but also thrombosis [53]. Thinning of the fibrous caps could occur in two possible ways: (1) the gradual loss of SMCs from the fibrous cap and (2) the degradation of the collagen-rich cap matrix by macrophages [54]. Ruptured fibrous caps contain fewer SMCs and less collagen than intact caps, and macrophage foam cells secrete proteolytic enzymes that degrade the fibrous cap, such as plasminogen activators, cathepsins, and matrix metalloproteinases [55]. A causal relationship between plaque rupture and thrombosis may exist, as dislodged plaques are often found within the thrombus. Plaque erosion is also often associated with thrombosis.

## 3. Anti-Inflammatory Effects of Exercise on Atherosclerosis

Because many existing therapeutics for atherosclerosis may not be completely effective and at most only delay the onset of atherosclerosis, there is a considerable interest in utilizing exercise as a possible non-pharmacologic treatment. Unlike anti-inflammatory therapeutics, physical exercise increases energy expenditure, promotes longevity, and helps patients reduce the risk for many chronic diseases [9]. As physical exercise can be used by many people in the general population, it should be promoted in the treatment of atherosclerosis.

Many studies and reviews have discussed numerous mechanisms that could facilitate the anti-inflammatory effects of exercise, such as the reduction of visceral fat, the reduction of pro-inflammatory monocytes and Toll-like receptors (TLRs) on monocytes and macrophages, and the increased production of anti-inflammatory exerkines from skeletal muscles and other secretory organs [56,57,58]. These mechanisms could be applied to the treatment of atherosclerosis, as atherosclerosis has many features similar to those of other chronic inflammatory diseases, such as Type 2 diabetes and RA. We thus devote this section to briefly discuss the mechanisms that bring about the anti-inflammatory effects of exercise. Major anti-inflammatory exerkines that could serve as potential therapeutic targets for atherosclerosis are discussed in the next section.

### 3.1. Reduction of Visceral Fat

Increases in visceral fat have been associated with increased mortality and risk of many diseases, including atherosclerosis and Type 2 diabetes. As physical exercise brings about a reduction in visceral fat and increases energy expenditure, it subsequently results in lower production of pro-inflammatory adipokines, including tumor necrosis factor (TNF), leptin, retinol-binding protein 4, lipocalin 2, interleukin-6 (IL-6), interleukin-18 (IL-18), CC-chemokine ligand 2 (CCL2 or MCP-1), CXC-chemokine ligand 5, and angiopoietin-like protein 2 and an increase in anti-inflammatory cytokines, such as adiponectin and secreted frizzled-related protein 5 [9,59]. Through a decrease in pro-inflammatory adipokines and fat deposits, exercise brings about a decrease in atherosclerotic risk and systemic inflammation.

### 3.2. Reduction of Pro-Inflammatory Monocytes and Regulatory T Cells in Circulation

There are two major types of monocytes: classical (CD14^hi^CD16^−^) and non-classical (CD14^low^CD16^+^ or CD14^hi^CD16^+^) monocytes, with the major difference being that inflammatory CD14^low^CD16^+^ monocytes express about 2.5 times more TLR4 on the cell surface than the other types of monocytes [60]. Despite constituting 10% of all monocytes [61], inflammatory monocytes could play a critical role in the inflammation of many diseases, including atherosclerosis and diabetes, due to their heightened antigen-presenting ability and capability to secrete higher levels of pro-inflammatory cytokines [13]. While short, individual bouts of exercise have been shown to have little effect on the number of inflammatory monocytes [62], regular exercise appears to reduce the proportion of inflammatory monocytes in the circulation. A cross-sectional analysis of age-matched healthy, physically inactive elderly men and women and physically active subjects who had 12 weeks of exercise demonstrated a 64% decrease in the percentage of inflammatory monocytes in physically inactive subjects following the training intervention [63]. In addition, high-intensity aerobic exercise could elevate regulatory T cell levels, reduce pro-inflammatory cytokines, and increase anti-inflammatory cytokine levels [9,64]. Acute and chronic exercise has also been found to downregulate TLR1, TLR2, and TLR4 expression on these monocytes. Specifically, acute exercise has been found to reduce TLR4 expression by 32% after one and a half hours of cycling and TLR2 expression by 12% only on pro-inflammatory monocytes [65]. Results from a 12-week chronic exercise training program also demonstrated TLR4 expression on inflammatory monocytes was significantly lower in younger and older participants [66].

### 3.3. Increased Levels of NO

Physical exercise could also exert anti-inflammatory effects by increasing NO levels. Mice studies have suggested that in hyperlipidemic mice, moderate aerobic exercise increases plasma levels of NO and endothelial NO synthase expression through murine macrophages, which can confer vascular protection and scavenge ROS [67,68]. However, prolonged strenuous physical exercise can reduce cyclic pulsations, thereby limiting NO production [67,68]. Therefore, moderate exercise should be used in the prevention of atherosclerosis, as it increases NO levels.

### 3.4. Inhibition of Monocyte and Macrophage Infiltration into Adipose Tissue

Another possible mechanism that facilitates the anti-inflammatory effects of exercise is the inhibition of monocyte and macrophage infiltration into adipose tissue. Macrophages and T cells that infiltrate adipose tissue are known to regulate its inflammatory state [69], and CCL2 and CCL3 may stimulate the recruitment of macrophages [70]. Therefore, the migration of peripheral blood mononuclear cells to inflamed sites may play a role in the development of atherosclerosis [71]. Mice studies have also demonstrated that exercise could decrease the expression of ICAM-1 in tissues, which is known to have a role in the adhesion of inflammatory cells to vascular endothelium and mediates interactions between T cells and target cells [72]. ICAM-1 expression increases with obesity in humans [72], and blocking its expression in obese mice prevented macrophage infiltration into adipose tissue [72]. Therefore, ICAM-1 may have a role in the reduction of macrophage infiltration into adipose tissue.

### 3.5. Phenotypic Switching of Macrophages Within Adipose Tissue

Phenotypic switching of macrophages within adipose tissue could also facilitate the anti-inflammatory effects of exercise. Animal studies have demonstrated inflamed adipose tissue is associated with the recruitment of M1-type macrophages or a phenotypic switch from M1- to M2- type macrophages [73]. M-1, or classically activated, macrophages produce pro-inflammatory molecules, such as TNF, IL-6, inhaled NO (iNO), and NO, and are induced by cytokines released by Type-1 helper T (Th1) cells, such as INF-γ, and TLR ligands 159]. They also produce interleukin-12 (IL-12), which is critical for the expansion of Th1 cells and the synthesis of INF-γ. M-2, or alternatively activated, macrophages produce anti-inflammatory cytokines, decrease the production of inflammatory cytokines, and up-regulate the expression of arginase 1, mannose receptor 1, and interleukin-1 receptor antagonist (IL-1ra) [59]. They also synthesize IL-12, which stimulates the expansion of Type-2 helper T (Th2) cells and the expression of IL-4 and interleukin-13 (IL-13). Rodent studies have also demonstrated chronic exercise could downregulate the expression of the M1-specific marker CD11c and TL4 in adipose tissue, which could trigger phenotype switching from the M1- to M2-phenotype [58]. Similar conclusions were attained in a human exercise study that demonstrated a rise in expression of the M2-specific markers CD14, AMAC1, and MR, and a decline in the expression of the M-1 specific markers IL-6, TNF-α, and MCP-1 [74]. Collectively, these findings suggest that regular exercise might favor the M-2 macrophage phenotype.

### 3.6. Enhanced Secretion of Glucocorticoids and Catecholamines

Physical exercise could also induce cortisol production from cholesterol, as its effects are transmitted to the hypothalamus that promotes the production of ACTH in the adrenal gland [75]. In addition to preserving blood pressure, cortisol could produce glucose based on proteins and promote lipometabolism and muscle function [76]. Adrenaline and noradrenaline are also produced by the adrenal medulla in response to exercise. Collectively, these glucocorticoids could block the synthesis of pro-inflammatory cytokines, such as IL-12 and TNF-α, by binding to their receptors on monocytes and macrophages and inducing a shift from a TH1 cell to a humoral Th2 immune response [77]. IFN-γ and IL-2 levels subsequently decline while interleukin-4 (IL-4) and interleukin-10 (IL-10) levels increase, even though they do not directly influence IL-10 production [77]. Catecholamines, such as epinephrine and norepinephrine, directly promote the synthesis of IL-10 and hinder IL-12 production without directly influencing Th2 cells [77,78].

## 4. Anti-Inflammatory Exerkines in the Treatment of Atherosclerosis

Another major mechanism that facilitates the anti-inflammatory effects of exercise is the release of anti-inflammatory exerkines into the circulation. Since the discovery that circulating cytokines and humoral factors are secreted not only by skeletal muscle but also by other secretory organs, there has been considerable interest in exploiting their therapeutic potential, as they could be used in many patient populations. In this section, although we define these exerkines to originate from any secretory organ, many of the potential anti-inflammatory exerkines for atherosclerosis that have available data to date are mostly secreted by skeletal muscle and adipose tissue. While there could be other exerkines that have anti-atherogenic effects, such as IL-10, they were not mentioned here as the exerkine field is still rapidly expanding and little data is currently available regarding their abilities to facilitate the effects of exercise. Where available, the intensity, type, and duration of exercise are also considered in this section.

### 4.1. Irisin

Irisin, a 12,587 Da hormone-like polypeptide with 112 amino acids, is synthesized in muscle tissue after the proteolytic cleavage of its precursor, the membrane protein fibronectin type III domain-containing protein 5 (FNDC5). With an N-terminal fibronectin type III-like domain that forms a continuous intersubunit β-sheet dimer and a flexible C-terminal tail, it is prone to dimerization without the influence of glycosylation, suggesting irisin activates its receptor through the irisin domain as a myokine ligand or as a paracrine or autocrine dimerization module on FNDC5-like receptors [79]. Many studies have demonstrated irisin stimulates the browning of white adipocytes, which increases their metabolic rates [80,81,82]. This finding led many investigators to hypothesize irisin levels would be lower in individuals with metabolic disorders. Indeed, this was confirmed in many clinical studies comparing patients with Type 2 diabetes, insulin resistance, and other metabolic syndromes with controls. Additionally, irisin improves energy consumption and at pharmacological concentrations (50–100 nmol/L), it enhances the proliferation capacity of hippocampal neurons and reduces their damage from neurodegenerative diseases [83]. As atherosclerosis has many features similar to those of other chronic inflammatory disorders, recent results from a carotid partial ligation model of apolipoprotein E (ApoE)-deficient mice fed on a high-cholesterol diet demonstrated irisin significantly suppressed carotid neointima formation and promoted human umbilical vein endothelial cell survival by upregulating microRNA126-5p expression through the ERK signaling pathway. Blocking microRNA126-5p expression subsequently abolished the inhibitory effects of irisin on neointima formation, lesional lipid deposition, macrophage area, and the endothelial cell proliferation effects [82,84]. Results from a separate study utilizing ApoE-deficient mice fed on a high-cholesterol diet and a mouse carotid partial ligation model also demonstrated irisin significantly reduced the severity of aortic atherosclerosis and restored ox-LDL-induced human umbilical vein endothelial cell dysfunction by inhibiting the reactive oxygen species (ROS)/p38 MAPK/NF-κB signaling pathway cell apoptosis through the upregulation of Bcl-2 and downregulation of Bax and caspase-3 expression [85].

Given its clear anti-inflammatory role in atherosclerosis, multiple studies have attempted to establish a relationship between exercise and irisin levels. Findings from a systematic review and multiple animal and human randomized controlled trials suggested although single bouts of exercise (sessions were about 30 to 45 min each) led to transient increases in plasma irisin levels, chronic exercise (3 to 4 sessions for 8 to 12 weeks) may actually reduce plasma irisin levels [86,87,88,89]. This parallels the idea that irisin is a metabolic stress-induced chemokine that transiently increases in response to cellular energy demand [90]. Even though higher intensity exercise may lead to higher irisin levels, with absolute workload exerting the great effect, this effect is transient and might explain irisin’s quick return to baseline levels. In fact, a recent study suggested irisin levels remained elevated above baseline for 125 min after exercise for moderate intensity whereas they returned to baseline just within 15 min post-exercise for high intensity [91]. Furthermore, resistance exercise appears to induce a greater increase in irisin levels compared to endurance exercise or a combination of them [91]. Although these findings suggest that acute resistance exercise at moderate intensity is most likely to induce higher irisin levels to exert the anti-inflammatory effects of exercise for atherosclerotic patients, further studies are needed to confirm these findings.

### 4.2. Adiponectin

Human adiponectin contains 244 amino acids and has a collagen-like and a globular C1q-like domain. It has at least three homomeric complexes: trimer, hexamer, and higher order multimers [92]. Plasma levels of adiponectin are inversely correlated with body mass index (BMI), abdominal fat, and weight gain [93]. Through activation of its two receptors, adiponectin receptor 1 (AdipoR1) and adiponectin receptor 2 (AdipoR2), it stimulates insulin secretion, decreases hepatic glucose production, and enhances glucose and fatty acid oxidation in skeletal muscles [94,95]. In various animal model and LPS-stimulated porcine macrophages and tissue experiments, adiponectin has been shown to inhibit the production of pro-inflammatory cytokines, including TNFα, IL-6, and IL-1β, and increase the production of IL-10 [96,97,98]. It also increases the effect of NO by activating the AMPK signal pathway through the AdipoR1 and AdipoR2 receptors, thus favoring vasodilation and inhibiting C-reactive protein (CRP) production, platelet aggregation, monocyte adhesion, and smooth muscle proliferation. Additionally, it downregulates the expression of scavenger A receptors and inhibits the conversion of macrophages into foam cells [99]. Through the COX-2-mediated anti-inflammatory and AMPK-mediated anti-apoptotic mechanisms, it also protects the heart from myocardial ischemia-reperfusion injury. Additionally, it attenuates ox-LDL and hyperglycemia-induced ROS generation by activating the cAMP7PKA cascade, reduces atherosclerotic plaque in the abdominal aorta, and blocks the transformation of macrophages to foam cells [100,101,102].

Due to its clear anti-inflammatory role in atherosclerosis, many studies have attempted to determine the association between exercise and adiponectin levels. Findings from a systematic review and multiple human and animal exercise experiments demonstrated adiponectin levels seem to significantly increase in both obese and healthy subjects after acute (between 30 and 60 min-sessions) and chronic exercise (2 to 4 sessions for 4 to 52 weeks) training, with high-intensity training (75–85% VO_2_ peak) generating a greater increase than low-intensity training (50% VO_2_ peak) [98,103,104]. High-intensity exercise might likely increase adiponectin levels, as results from studies that set exercise protocols at low to moderate intensity (<65% VO_2max_) showed no change in adiponectin levels [96,97,105]. Scarce data is available regarding the effects of resistance training in adiponectin levels; however, de Mello et al. suggested a combination of resistance and endurance exercise could generate a significantly greater increase in adiponectin levels compared to endurance training alone [106]. Together, these findings suggest that high-intensity acute or chronic exercise is most likely to generate higher levels of adiponectin; however, due to the scarcity of data and the low statistical power of available studies, more investigation is needed in this area.

### 4.3. IL-6

Human IL-6 consists of 212 amino acids and has a four-helix bundle structure containing four long α-helices arranged in an up-up-down-down topology. It is part of a family of cytokines that plays an important role in immune reactions, metabolic processes, and hematopoiesis [107]. All IL-6-related cytokines, except for IL-31, have the membrane glycoprotein gp130 as a common receptor and signal transducer subunit. The IL-6 receptor (IL-6R) also has an 80 kDa type 1 cytokine α-receptor subunit that is largely expressed in hepatocytes, leukocytes, and megakaryocytes [74]. Unlike common pro-inflammatory cytokines, such as interleukin-1 (IL-1) and TNFα, IL-6 does not promote major inflammatory mediators [108], and is thus known as a pleiotropic cytokine. Additionally, IL-6 initiates many anti-inflammatory hepatocyte-derived acute-phase proteins and inhibits the expression of IL-1 and TNFα and induces IL-1ra and IL-10 after exercise [109].

Although IL-6 has both pro-inflammatory and anti-inflammatory effects, these effects are mediated by two different signaling pathways: classic-signaling and trans-signaling. Whereas classic-signaling involves IL-6 binding to the membrane-bound non-signaling α-receptor IL-6R, leading to dimerization and activation of gp130, trans-signaling involves IL-6 binding to a soluble form of IL-6R (sIL-6R) that eventually forms a complex to activate gp130 [110]. This distinction is important, as cells that do not express membrane-bound IL-6R can still be activated by the IL-6/sIL-6R complex. In addition, trans-signaling allows lymphocyte trafficking into an inflamed area by controlling chemokine expression, promoting T-cell proliferation during colon cancer development, and regulating expression of adhesion molecules on endothelial cells [111]. Unlike classic-signaling, which is important for controlling homeostatic processes and immunological outcomes [112], trans-signaling is important for the recruitment and apoptosis of leukocytes, maintaining the effector function of T cells, and the inflammatory activation of stromal tissues [113]. Murine sepsis models and animal experiments have demonstrated the pro-inflammatory effects of IL-6 are dependent on trans-signaling whereas the anti-inflammatory effects of IL-6 depended on classic-signaling [114,115]. For atherosclerosis, mice models and human macrophage experiments demonstrated a lifetime IL-6 deficiency enhances atherosclerotic plaque formation in ApoEK^−/−^–IL-6^−/−^ mice, leading to worse vascular development processes [116], and that IL-6 enhanced the capacity of macrophages to ingest apoptotic cells and attenuated the pro-inflammatory phenotype of cholesterol-loaded macrophages [117]. Multiple reviews and studies demonstrated blocking IL-6 trans-signaling through the circulating soluble glycoprotein 130 (sgp130) prevented high-fat diet-induced macrophage recruitment into adipose tissue, and inhibited mononuclear cell-dominated inflammatory processes [118,119]. Sgp130 treatment has also been shown to decrease endothelial activation and intimal SMC infiltration, and reduce monocyte recruitment and atherosclerotic progression in hypercholesterolemic Ldlr^−/−^ mice [120]. Treatments that target site IIb of the IL-6 receptor has also been shown to selectively block trans-signaling [121]. However, the association of sgp130 levels and CVD appears less straightforward in human studies, with many reporting an inverse, null, or positive association [122]. These differences could be attributed to the notion that higher sgp130 levels reflect higher fragility rather than a cause of adverse outcome of the subjects [123]. Nevertheless, promoting the classic-signaling pathway and inhibiting the trans-signaling pathway of IL-6 may have a protective effect for atherosclerotic patients.

Due to the potential atheroprotective effects of IL-6, many studies have attempted to establish a relationship between exercise and IL-6 levels. Findings from multiple reviews and human exercise experiments have demonstrated IL-6 significantly increases after 30 min and peaks at the end of 2.5 h of treadmill running [124,125,126,127]. These levels can increase up to 100-fold in an exponential manner after a 3–3.5 h marathon [128,129]. However, IL-6 levels rapidly decline, with a half-life of one to two hours. Additionally, IL-6 levels seem to correlate positively with exercise intensity. In fact, in a recent study involving ten healthy and active participants, those with high-intensity cycling training (5 × 4 min intervals at 80% VO_2max_ interspersed with 3 min intervals at 50% VO_2max_) had a 2.7-fold increase in IL-6 while those with low-intensity training (35 min cycling at 50% VO_2max_) only had a 1.4-fold increase in IL-6 [130]. Scarce data is available regarding the relationship between different modes of exercise and IL-6 levels. However, a systematic review suggested, in an analysis of 67 exercise trials, while the mode of exercise does not appear to have an effect on IL-6 levels, protocols using running as the source of exercise seem to have the greatest increase in IL-6 [131]. Although more investigation is required to validate these results, these findings suggest longer periods of high-intensity running may lead to higher IL-6 levels and more anti-inflammatory benefits for atherosclerotic patients.

### 4.4. IL-1ra

IL-1β is widely recognized as a pro-inflammatory cytokine for atherosclerosis, as its systemic increase subsequently transforms the disease caused by a simple accumulation of lipids into a disorder with complex inflammatory events in the arterial wall [132]. With 12 antiparallel beta-strands arranged into a beta-trefoil, it is involved in a wide range of inflammatory processes in atherosclerosis, including structural and functional alterations on cardiac myocytes, regulation of MAP kinase and iNO pathways, induction of adrenomedullin and VEGF, vessel wall inflammation, leukocyte chemotaxis and adhesion, monocyte infiltration into the subendothelial space, and upregulation of matrix metalloproteinases to promote plaque instability [132]. To abolish these inflammatory effects, the most widely investigated inhibitor of IL-1β is the IL-1 receptor antagonist (IL-1ra). Although it has both soluble and intracellular isoforms, the soluble form binds to the IL-1 receptor to facilitate its anti-inflammatory activity and the intracellular form has been shown to interact with the third component of the COP9 signalosome CSN3 and inhibit the activity of CSN-associated kinases as well as IL-1-mediated release of IL-6 and IL-8 [133,134]. In multiple reviews and animal studies, administering IL-1ra or antibodies that target IL-1β has been shown to inhibit many atherogenic cytokines from endothelial cells and SMCs, such as IL-6, IL-8, IL-4, IL-7, MCP-1, and TNF α, regulate matrix metalloproteinases of the extracellular matrix, such as MMP-13, inhibit vascular SMC proliferation, decrease foam-cell lesion size, improve outcomes in acute MI and ischemic stroke, and modulate plaque composition in ApoE^−/−^ mice [135,136,137,138,139,140,141]. IL-1ra^−/−^ mice also appear to be enriched in macrophages, depleted in SMCs, and develop fattier livers and hypercholesteroremia [134,142]. Additionally, endothelial cells lacking IL-1ra have been shown to have a reduced lifespan and growth compared to controls [134].

Many studies have attempted to establish a relationship between exercise and IL-1ra levels. IL-1ra levels appear to markedly increase after a marathon race, and repeated bouts of prolonged cycling at 75% VO_2max_ levels provoked more pronounced increases in plasma IL-1ra levels in elite athletes [143,144]. IL-1ra levels increase further two hours after a marathon session, demonstrating they peak 1.5 to 2 h after exercise [124,125,126]. Resistance training has also been shown to increase IL-1ra levels, with only the highest intensity (10 to 12 repetitions at 80% of 1RM) corresponding to a significant increase in IL-1ra levels [145,146,147]. Although these studies are limited by their small statistical power, these results suggest that repeated bouts of high-intensity endurance or resistance exercise could have more therapeutic potential for treating atherosclerosis.

## 5. Efficacies of Existing Anti-Inflammatory Therapeutics in the Treatment of Atherosclerosis

Unlike physical exercise, many current anti-inflammatory therapeutics at most only delay the progression of atherosclerosis, may be used by only a certain group of the general population, and are still under development and evaluation, despite their long-standing recognition in the treatment of atherosclerosis. Because they have been approved for other conditions that have similar features to atherosclerosis, they could be used to treat atherosclerosis, and their therapeutic efficacies and adverse effects have been extensively reviewed elsewhere [8,148,149,150,151,152]. These therapeutics can be broadly divided into four categories: “classic” anti-inflammatory drugs, biological pathway therapies, lipid mediators, and intracellular pathway inhibitors. To provide further context for the therapeutic potential of anti-inflammatory exerkines in the treatment of atherosclerosis, we discuss a few examples of anti-inflammatory therapeutics in each category, and briefly summarize their limited efficacies and current development. Table 1 offers a more extensive overview of these therapeutics, and describes their limited efficacies and highest stage of clinical development.

### 5.1. “Classic” Anti-Inflammatory Drugs

For the past few decades, many conventional anti-inflammatory drugs have been evaluated to treat atherosclerosis. For instance, methotrexate, a folic acid antagonist, downregulates adhesion molecules, inhibits cyclooxygenases and lipoxygenases, and modulates the secretion of cytokines and matrix metalloproteinases [153]. Many meta-analyses have suggested methotrexate is associated with a reduction in CVD mortality and myocardial infarction (MI) in patients with rheumatoid arthritis (RA) and psoriasis [154,155]; however, the METIS trial demonstrated no reductions in CRP levels or adverse CVD events among patients with ischemic heart failure (despite showing an improvement in functional class (New York Heart Association)) [156]. The CIRT trial, a phase 3 clinical trial, is currently underway to confirm these results in patients with stable coronary artery disease (CAD) [157]. As another example, colchicine is used in the treatment of arthritis and gout, and has a crucial role in atherosclerosis, as it can inhibit crystal-induced activation from macrophages, which can decrease the secretion of IL-1β [41,158]. Many retrospective studies have suggested that colchicine treatment was associated with a decreased risk of MI [159,160]. Despite its poor design, the LoDoCo trial demonstrated colchicine was associated with a reduction of acute coronary syndrome (ACS), out-of-hospital cardiac arrest (OHCA), and non-cardioembolic ischemic stroke [161]. An alternative approach to inhibit inflammasome-dependent activation is to neutralize IL-1β; this is currently being evaluated by the CANTOS trial [162].

### 5.2. Biological Pathway Therapies

Recombinant proteins and monoclonal antibodies of cytokines have also been used to treat CVDs and other chronic inflammatory diseases. TNF inhibitors, such as etanercept, infliximab, and adalimumab, are commonly used in patients with RA, psoriasis, or other autoimmune diseases, as they have a considerably increased CVD risk. Geborek et al. and Barerra et al. demonstrated RA patients who received the anti-TNF therapy had a 0.46 rate of having a CVD event than those who did not, and had increased HDL levels and decreased CRP and IL-6 levels [167,168]. However, there is scarce clinical trial data on the efficacy of anti-TNF inhibitors. Another potential biological pathway therapy is IL-1 inhibitors, as in endothelial cells, IL-1 modulates hemostatic properties and leukocyte adhesion while it induces SMCs to produce prostanoids and IL-6 [225]. The MRC-ILA Heart Study demonstrated a significant decrease in CRP and IL-6 levels for patients with non-ST elevation ACS (NSTE-ACS) who had the IL-1 receptor antagonist treatment [169]. In a separate clinical trial that randomly allocated canakinumab, a human monoclonal antibody that neutralizes IL-1β, to 556 men and women with Type 2 diabetes and high CVD risk, those who received the treatment had a significant reduction in CRP, fibrinogen, and IL-6 levels without major changes in LDL or HDL levels [170].

### 5.3. Lipid Mediators

Lipid mediators, such as phospholipase A2 inhibitors (secretory PLA2 (sPLA2) inhibitors, lipoprotein-associated PLA2 (LpPLA2) inhibitors, nonsteroidal anti-inflammatory drugs (NSAIDs)), antileukotrienes, statins, and other LDL, triglyceride, and HDL lowering therapies, either inhibit lipid biosynthesis or various seven-transmembrane G-protein coupled receptors. An example of NSAIDs is COX-2 inhibitors, and they have favorable effects on inflammation, oxidative stress, endothelial function, and tissue factor expression, but are associated with increased CVD risk [97,98]. Celecoxib, a COX-2 inhibitor, has been associated with fewer CVD events compared to ibuprofen and naproxen in the PRECISION trial; however, this result was not significant [186]. Additionally, a recent study using a nationwide cohort of 28,947 patients with OHCA of whom 3376 patients were treated with an NSAID also found a significantly increased risk of OHCA among patients who used ibuprofen and diclofenac but not those who consumed naproxen, celecoxib, or rofecoxib [187]. Another lipid mediator is anti-leukotrienes, as arachidonic acid metabolism by arachidonate 5-lipoxygenase (5-LO) and 5-LO-activating protein (FLAP) generates leukotrienes that have atherosclerotic implications, including lesion development, induction and activation of leukocytes, SMC proliferation, and endothelial dysfunction [226]. Inhibiting 5-LO-activating protein (FLAP) in ApoE and Ldlr double-knockout mice could reduce atherosclerosis and blocking leukotriene receptors have beneficial effects [188]. Clinical trials have demonstrated a non-significant reduction in CRP in patients at risk for CAD treated with DG-031 (veliflapon) [189] and significantly reduced plaque volume in patients with ACS treated with the 5-LO inhibitor atreleuton just after 24 weeks [190]. Statins are another popular atherosclerotic therapeutic, as they could decrease cholesterol synthesis by inhibiting the 3-hydroxy-3-methylglutaryl-coenzyme A (HMG CoA) reductase enzyme that blocks mevalonate synthesis [36]. For pravastatin, the CARE study demonstrated post-MI patients who received the treatment had decreased CRP levels (but not cholesterol) [191] while the WOSCOP trial demonstrated it significantly reduced MI incidence and death from CVD causes without affecting the risk of death from non-CVD factors in men with moderate hypercholesterolemia with no history of MI [192]. For lovastatin, the AFCAPS/TexCAPS trial showed a significant reduction in serious CVD events in patients without a history of CVD while only patients with LDL levels <150 mg per dl and CRP levels >2 mg per liter had significant clinical benefit [193]. The 4S trial also demonstrated treatment with simvastatin improves survival among 4444 patients with angina pectoris or previous MI [194]. For atorvastatin, the PROVE IT-TIMI 22 trial demonstrated patients who received the treatment had a significant reduction in CRP and LDL levels than those who received the pravastatin treatment [195]. For rosuvastatin, the ASTEROID trial discovered very high-intensity statin therapy using 40 mg/day of rosuvastatin resulted in significant regression of atherosclerosis among 507 patients with intravascular ultrasound trials [196], the SATURN trial found that maximal doses of rosuvastatin and atorvastatin resulted in significant regression of coronary atherosclerosis [197], and the JUPITER trial demonstrated lowering LDL levels with statins would reduce adverse CVD events in patients with ≤130 mg per dL LDL levels [198]. LDL and triglyceride lowering therapies also gain considerable interest. For instance, fibrates, which are agonists of peroxisome proliferator-activated receptor α (PPAR-α), can lower triglycerides and triglyceride-rich lipoprotein particles up to more than 50% while increasing HDL levels [59]. However, results from several clinical trials and meta-analyses failed to demonstrate good CVD outcomes [201].

### 5.4. Intracellular Pathway Inhibitors

The final type of anti-inflammatory therapeutic for atherosclerosis are intracellular pathway inhibitors, which modulate intracellular inflammatory responses and include p38 mitogen-activated protein kinase (MAPK) inhibitors, statins, NADPH oxidase inhibitors, and phosphodiesterase (PDE) inhibitors. PDE inhibitors, such as PDE3, PDE4, and PDE5 inhibitors, are of interest because blocking phosphodiesterases, which degrade anti-inflammatory cyclic nucleotides, might enhance the effects of endogenous anti-inflammatory mediators. For example, cilostazol, a PDE3 inhibitor, has been shown to decrease atherosclerotic lesion size, reduce macrophage accumulation in ApoE knockout mice [218], and inhibit vascular SMC proliferation in vitro [148] and intimal hyperplasia in vivo [219]. In addition, clinical trial data suggest that cilostazol reduces restenosis [147]. Another potential intracellular pathway inhibitor is antioxidants. Succinobucol (AGI-1067), which is related to probucol, is the most promising therapy in this category [8]. Results from the ARISE clinical trial suggested that, among a group of 6144 patients who had MI or unstable angina within a year before recruitment, those who received succinobucol had a decrease in diabetic occurrences and fewer patients had fewer composite secondary end points (these include CVD death, cardiac arrest, MI, and stroke) [223]. In the CART-2 trial, succinobucol treatment had favorable effects on lumen dimensions in stented vessels and led to a significant reduction in plasma myeloperoxidase levels [224].

## 6. Challenges and Future Directions

Despite the anti-inflammatory benefits of exercise and exerkines, several challenges must be addressed before they are deployed in the clinic. One of the key challenges in exploiting the therapeutic potential of exerkines is to develop exerkine analogues that take into account their proteolytic instability, short half-lives, immunogenicity, and toxicity when administering them into patients. Recent reviews have suggested protein pharmaceuticals are viable options as currently there are two dominant technologies that have developed many drugs in the market or in clinical trials: PEGylation and Fc fusion [227,228]. As of 2016, there were 12 marketed PEGylated biopharmaceuticals [229]. Further work is needed in this area to determine other viable therapeutic options.

Another key challenge in exploiting the therapeutic potential of exerkines is to achieve tissue-specific targeting without having side effects. Although many exerkines are expressed in skeletal muscle or adipose tissue and are induced by muscle contraction, they are also expressed in other tissues and could generate side effects when they are being injected into a patient [228,230]. Recent findings have suggested that nanotechnology could be used to address this issue as nanoparticles can target specific tissues and can provide variable circulation times by controlling the target surface and size [231,232].

A final challenge in exerkine biology is to clarify the relationship between these anti-inflammatory exerkines with different types of exercise. As mentioned in earlier sections of this review, most of the studies to date have focused primarily on endurance exercise and while there are other anti-atherogenic exerkines, little data is available regarding their relationship with exercise. Additionally, due to the low statistical power of most available studies, many of the above findings need to be validated in larger populations while taking into account various health characteristics of these subjects. Although challenging, collectively, these findings will help elucidate the therapeutic potential of these exerkines in the treatment of atherosclerosis.

## 7. Conclusions

A growing body of evidence suggests that exercise and anti-inflammatory exerkines could have therapeutic potential in the treatment of atherosclerosis, as they have multisystemic, anti-inflammatory benefits. Unlike anti-inflammatory therapeutics, which at most only delay the progression of atherosclerotic disease and are still under evaluation, physical exercise increases energy expenditure, promotes longevity, and helps patients reduce the risk for many chronic diseases [9]. It also brings about many anti-inflammatory benefits, such as reducing visceral fat, decreasing pro-inflammatory monocytes and regulatory T-cells in the circulation, and inhibiting monocyte and macrophage infiltration into adipose tissue. Several exerkines are also potentially useful to treat atherosclerosis, as they have many benefits, including inhibiting monocyte adhesion and promoting endothelial cell survival potential. Current exerkine targets for atherosclerosis should include irisin, adiponectin, IL-6, and IL-1ra, among others, as they have the most promising results. While further studies are necessary to fully understand the relationship between these anti-inflammatory exerkines and exercise and develop viable therapeutic analogues for atherosclerotic patients, physical exercise and bioengineered exerkines should serve as potential therapeutic targets for the treatment of atherosclerosis in the future.

## Figures and Tables

**Table 1:** Current Anti-Inflammatory Therapeutics for Atherosclerosis.

Type of Therapeutic	Categories and Examples	Efficacy	Highest Stage of Clinical Development
“Classic” anti-inflammatory drugs	Methotrexate	Reduces CVD mortality and MI in patients with RA and psoriasis [154,155]; does not reduce CRP levels or adverse CVD events among patients with ischemic heart failure (METIS) [156]	Phase III
	Colchicine	Can inhibit crystal-induced activation from macrophages, which can decrease the secretion of IL-1β [41,158]; decreases MI risk [159,160]; reduces ACS, OHCA, and non-cardioembolic ischemic stroke (LoDoCo) [161]	Phase III
	Allopurinol	Improves endothelial function and reduces levels of oxidative stress, and could reduce atherosclerotic lesion size in ApoE knockout mice [163,164,165]; increases exercise tolerance, reduces vascular tissue oxidative stress, improves endothelial function, and lowers mortality among patients with CAD or heart failure [166]	Phase II
Biological pathway therapies	TNF-inhibitors (etanercept, infliximab, and adalimumab)	Lowers risk of having CVD, CRP, and IL-6 levels and increases HDL levels in patients with RA [167,168]; scarce data for atherosclerosis	Phase III
	IL-1 inhibitors (canakinumab)	Decreases CRP and IL-6 levels for patients with non-ST elevation ACS (NSTE-ACS) (MRC-ILA Heart Study) [169], and reduces CRP, fibrinogen, and IL-6 levels in patients with Type 2 diabetes and high CVD risk without major changes in LDL or HDL levels [170]	Phase III
	Anti-IL-12-subunit p40 inhibitors (ustekinumab and briakinumab)	Similar odds ratio for CVD in patients treated with ustekinumab or briakinumab compared to placebo, but a higher odds ratio for CVD for patients who received both treatments [171]	Phase III
	Chemokine signaling therapy	MLN1202, a MCP-1 inhibitor, significantly decreases CRP levels in participants with two or more risk factors for atherosclerosis [172]; a CCL17-blocking antibody was developed that can limit the expansion of regulatory T-cells, but no clinical data is available [173]	Phase II
Lipid mediators	sPLA_2_ inhibitors (most advanced is varespladib)	Significantly reduces the level of atherosclerotic lesions when given alone or with a statin in mice studies [174,175]; significantly reduces LDL, CRP, and sPLA2-IIA levels and the number of atherosclerotic lesions with atorvastatin (ACS-FRANCIS) [176]; associated with an increased risk of MI and composite secondary end point of CVD mortality, MI, and stroke (VISTA-16) [177]; certain sPLA2 isoforms may have different atherosclerotic properties in mice studies and thus lead to varying atherosclerotic lesion sizes [178]	Phase III
	LpPLA_2_ inhibitors (most advanced is darapladib)	Reduces LpPLA2 activity in plasma and arteries, decreases plaque area in the left anterior descending coronary artery, and reduces the necrotic core area [179]; reduces necrotic core growth but not coronary plaque volume (IBIS-2) [180]; no beneficial effects in preventing the primary endpoint of CVD, MI, or stroke in 15,828 patients with CAD (STABILITY) [181]; no reduction in the risk of major coronary events in 13,026 patients with ACS (SOLID-TIMI 52) [182]	Phase III
	NSAIDs	COX-1: aspirin could lead to irreversible inhibition of COX-1 in platelets [183]; high doses of aspirin could inhibit COX-2 and prevent inflammation-mediated endothelial dysfunction and release IL-7 effects while lead to more GI side effects [184]; terutroban was not superior to aspirin and could lead to minor bleeding in a follow-up of 3.5 years in >19,000 patients with previous ischemic stroke or transient ischemic attack (PERFORM) [185] COX-2: associated with increased CVD risk that led to their withdrawal [97,98]; celecoxib demonstrated an insignificant number of fewer CVD events compared to ibuprofen and naproxen (PRECISION) [186]; significantly increased risk of OHCA among patients who used ibuprofen and diclofenac but not those who consumed naproxen, celecoxib, or rofecoxib [187]	Marketed (for aspirin)
	Anti-leukotrienes	Inhibits FLAP in ApoE and Ldlr double-knockout mice [188]; reduces atherosclerosis and blocking leukotriene receptors have beneficial effects [188]; nonsignificant reduction in CRP for patients at risk for CAD treated with DG-031 (veliflapon) [189]; significantly reduced plaque volume just after 24 weeks for ACS patients who received the 5-LO inhibitor atreleuton treatment [190]	Phase III
	Statins	Decreases cholesterol synthesis by inhibiting the 3-hydroxy-3-methylglutaryl-coenzyme A (HMG CoA) reductase enzyme [36]; for pravastatin, post-MI patients who received the treatment had decreased CRP levels (but not cholesterol) (CARE) [191] while men with moderate hypercholesterolemia with no history of MI had significantly reduced MI incidence and death from CVD causes without affecting the risk of death from non-CVD factors (WOSCOP) [192]; for lovastatin, patients without a history of CVD had a significant reduction in serious CVD events in and only patients with LDL levels <150 mg per dL and CRP levels >2 mg per liter had significant clinical benefit (AFCAPS/TexCAPS ) [193]; for simvastatin patients with angina pectoris or previous MI had improved survival (4S) [194]; for atorvastatin, patients who received the treatment had a significant reduction in CRP and LDL levels than those who received the pravastatin treatment (PROVE IT-TIMI-22) [195]; for rosuvastatin, 40 mg/day doses resulted in significant regression of atherosclerosis among 507 patients with intravascular ultrasound trials (ASTEROID) [196], maximal doses of rosuvastatin and atorvastatin resulted in significant regression of coronary atherosclerosis (SATURN) [197], and lowering LDL levels with statins would reduce adverse CVD events in patients with ≤130 mg per dl LDL levels (JUPITER) [198]	Marketed
	Cholesterol absorption inhibitors (ezetimibe)	Reduces LDL by 15–22% alone and 15–20% when combined with a statin; clinical trials support its administration as second-line therapy in statin-intolerant patients or in patients with contraindication to statins (PRECISE-IVUS; IMPROVE IT) [199,200]	Phase III
	Bile acid sequestrants (cholestyramine, colestipol, and colesvelam)	Reduce LDL levels of 18–25% at the highest dose [201]; cholestyramine and colestipol have been found to have GI adverse effects and major interactions with other drugs, which limit their use [59]	Phase III
	Proprotein convertase subtilisin/kexin type-9 (PCSK-9) inhibitors	Lower LDL levels more than ezetimibe combined with statins as they can prevent PCSK-9 from binding to LDL receptors in the liver and stimulating LDL absorption and degradation of these receptors [59]; clinical trials have demonstrated that evolocumab and alirocumab could decrease atheroma volume and CVD events [202,203,204]; PCSK-9 inhibitors are still limited by their relatively high costs [59]	Phase III
	Fibrates	Lower triglycerides and triglyceride-rich lipoprotein particles up to more than 50% while increasing HDL levels [59]; clinical trials and meta-analyses failed to demonstrate good CVD outcomes [205]	Phase III
	n-3 fatty acids (eicosapentaenoic acid and docosahexaenoic acid)	Lower triglyceride levels up to 45% [206]; no significant effect of ω-3 fatty acids on composite CVD events [207]	Phase III
	Niacin	Could reduce CVD events and adverse coronary endpoints; clinical trials have demonstrated niacin did not lead to a reduction in CVD events and may even cause severe side effects (AIM-HIGH; ACCORD; HPS2-THRIVE) [34]	Phase III
	Cholesteryl ester transfer protein (CETP) inhibitors	Could reduce LDL levels and increase HDL levels, but clinical trials have failed to determine their efficacies (ILLUMINATE; Dal-OUTCOMES; ACCELERATE); only anacetrapib is still actively investigated, as it could decrease LDL levels [208]	Phase III
Intracellular pathway inhibitors	p38 MAPK inhibitors	p38 inhibitor SB203580 treatment had attenuated atherosclerosis [209]; dilmapimod could lead to lower levels of CRP [210]; losmapimod could improve endothelial function just after four weeks or decrease CRP levels in patients with hypercholesterolemia or had a history of atherosclerosis (SOLSTICE) [211,212,213,214]	Phase II
	NADPH oxidase inhibitors	Nox1 or Nox2 deletion in ApoE knockout mice reduces atherosclerotic lesion size and correlates with lower levels of aortic superoxide [215,216]; the Nox1/Nox4 inhibitors GKT136901 and GKT137831 do not completely suppress ROS production but could slow or prevent disease progression [217]	Phase II
	PDE (PDE3, PDE4, PDE5) inhibitors	PDE3 inhibitors: Cilostazol decreases atherosclerotic lesion size, reduces macrophage accumulation [218], and inhibits vascular SMC proliferation in vitro [148] and intimal hyperplasia in vivo [219]; reduces restenosis [122] PDE4 inhibitors: Roflumilast decreases cytokine and adhesion molecule expression [220]; decreases the rate of CVD in chronic obstructive pulmonary disease (COPD) patients [221] PDE5 inhibitors (sildenafil, vardenafil, and tadalafil): sildenafil could restore endothelial function and slow atherosclerosis development in ApoE knockout mice [222]	Phase IV
	Antioxidants (most advanced therapeutic is succinobucol)	Decreases diabetic occurrences and composite secondary end points (these include CVD death, cardiac arrest, MI, and stroke) in patients who had MI or unstable angina (ARISE) [223]; improves effects on lumen dimensions in stented vessels and leads to a significant reduction in plasma myeloperoxidase levels (CART-2) [224]	Phase III
